# Antibody screening data of human midgestation liver cells with a focus on hematopoietic, liver sinusoidal endothelial, and hepatoblast cell-populations

**DOI:** 10.1186/s13104-022-06229-3

**Published:** 2022-12-06

**Authors:** Marcus O. Muench, Christopher Nosworthy

**Affiliations:** 1 Vitalant Research Institute, 360 Spear Street, Suite 200, 94105 San Francisco, CA USA; 2 Department of Laboratory Medicine, University of California, 94141 San Francisco, CA USA

**Keywords:** Flow cytometry, Antigens, Hepatology, Liver, Endothelial Cells, Blood Cells, Leukocyte Common Antigens, Human, Fetus

## Abstract

**Objectives:**

Cell-surface antigen screening was performed on human fetal liver cells using flow cytometry. The goal was to provide proteomic expression data on a number of human fetal liver cell populations that can inform studies on developmental hepatology and hematology.

**Data description:**

A 21 weeks’ gestation liver was depleted of erythrocytes prior to antibody staining. Screening was performed using phycoerythrin-labelled antibodies against 332 antigens. In addition to these antibodies, all samples were stained for CD14, CD45, CD235a, and CD326 (epithelial cell adhesion molecule – EpCAM). Subpopulations of fetal liver cells were identified using the co-stained antigens. Hematopoietic cells were identified by their expression of CD45 and CD235a; non-hematopoietic cells were further subdivided based on CD14 and CD326 expression. CD326^++^CD14^low^ hepatoblasts and CD14^++^ liver sinusoidal endothelial cells were analyzed for the frequency and intensity of antigen expression. Analyzed flow cytometry data are presented for the expression of the antigens on hematopoietic cells and on non-hematopoietic cells in the context of CD14 and CD326 expression.

## Objective

During prenatal development the cellular composition of the liver is complex as the liver is a major site of hematopoiesis while also supporting the development of the hepatocytic elements that dominate the adult liver [1]. Moreover, there are notable differences between adult hepatocytes and their prenatal precursors known as hepatoblasts. To better distinguish and isolate different cell types for study, flow cytometric screening of cell surface antigens was performed to provide phenotype data for multiple cell types found in the human midgestation liver. One aim was to discover new antigens that are useful in distinguishing cell types, but confirmation of antigen expression only known from studies of human adult liver or liver cells of other species was also sought.

Five color flow cytometry was used to identify different subsets of live cells using three colors in addition to the antibody screening performed using phycoerythrin (PE) labeled antibodies. Hematopoietic cells were stained with a combination of CD45 to identify progenitors and mature leukocytes as well as CD235a to mark any remaining erythrocytes not depleted from the samples. CD326, although expressed by many different cell types in the fetal liver [2], was used to identify hepatoblasts [3, 4]. CD14 expression further distinguished hepatoblasts as well as liver sinusoidal endothelial cells (LSECs) [2, 5].

These data may be of value to researchers studying liver ontogeny, normal development of hepatocytic cells, and the development of liver cancer. This work provides a resource for protein expression data for researchers studying human developmental hepatology, hematology, immunology, and liver sinusoidal endothelial cell biology. The authors have submitted a manuscript that further evaluates these data on hepatoblasts, LSECs, and hematopoietic cells [6].

## Data description

Cells were analyzed using an LSR II flow cytometer (BD) (Table 1) and data analyzed using FlowJo software, version 9 (FlowJo, Inc.) for presentation [7]. The number of events collected was maximized to provide the largest possible number of events for analysis and ranged from 3.33 × 10^4^ to 2.80 × 10^5^ events/sample, with a mean of 1.06 × 10^5^ events. This sometimes resulted in drop-off in flow rate at the end of sample collection or a rapid increase owing to air bubbles. This can be mitigated using the time parameter to select for analysis only events collected from a period of stable flow rate (Fig. 1 A – all figures stored in the data repository listed in Table 1). Live cells, lacking propidium iodide staining (Fig. 1B), can be selected and further gated to reduce the number of doublet events (Fig. 1 C) and small debris (Fig. 1D) based on light scatter measurements to improve the quality of data for analysis.

As the fetal liver is primarily a hematopoietic organ, the cells were stained with a combination of CD45 and CD235a antibodies to identify blood cells (Fig. 1E). Before staining, the liver cells were depleted of erythrocytes by immunomagnetic bead depletion [2, 5] but as this depletion is not fully effective – specially with immature erythrocytes that express low levels of CD235a – additional staining with CD235a was performed to better differentiate the hematopoietic from the non-hematopoietic fraction. Cells stained with fluorochrome-labelled non-specific IgG1 antibodies can be used to guide the placement of gates as shown in Fig. 2 A-B.

Samples were stained with CD14 and CD326 to differentiate subpopulations of non-hematopoietic cells (Fig. 1 F). High levels of CD326 expression and low levels of CD14 expression can be used to identify fetal hepatoblasts (Fig. 1 F and 2 C) [2–4]. Liver sinusoidal endothelial cells (LSECs) can be gated based on high expression levels of CD14 [5]. The different light scatter properties of hematopoietic cells (Fig. 1G), hepatoblasts (Fig. 1 H), and LSECs (Fig. 1I) further indicate differences in the size and complexities of these cells. Using the common markers stained in all the samples, it is possible to further identify other subpopulations of cells beyond those described.

Collection of data using the flow cytometer took place over 19.5 h: the first of four 96-well sample plates was completed in just over 3.5 h, followed by a break in analysis for 6.7 h, and then the 3 remaining plates were analyzed in succession over 3.5 h, 3.4 h, and 2.1 h for the partially filled fourth plate. Data analysis revealed some shifts in the frequencies of cell populations over the course of the experiment (Fig. 3). Notably, the hepatoblast population was negatively affected by the prolonged period of cold storage.

The complete list of antigens and antibody clones are indicated in Fig. 4. Analysis of the antigens in the context of CD45/CD235a expression is shown in Fig. 5. In Fig. 6, the expression of the antigens is shown on the non-hematopoietic cell fraction using polychromatic plots to indicate CD14 expression as shown in Fig. 1 J. The frequency of antigen expression as well as the mean fluorescence intensity of antigen expression for gated CD45/CD235a^+^ hematopoietic cells (Fig. 1E), CD326^++^CD14^low^ hepatoblasts, and CD14^++^ LSECs (Fig. 1 F) are listed in Fig. 4.

**Table 1:** Overview of data files/data sets

Label	Name of data file/data set	File types (file extension)	Data repository and identifier (DOI or accession number)
Data set 1	Human 21 week gestation liver phenotype	FCS files (.fcs)	FlowRepository ID: FR-FCM-Z58Z (https://flowrepository.org/id/FR-FCM-Z58Z) [7]
Data File 1	Tissue_and_methods	PDF file (.pdf)	FlowRepository ID: FR-FCM-Z58Z (https://flowrepository.org/id/FR-FCM-Z58Z) [7]
Data File 2	_All_Figures	PDF file (.pdf)	FlowRepository ID: FR-FCM-Z58Z (https://flowrepository.org/id/FR-FCM-Z58Z) [7]

## Limitations

- The data are derived from a single midgestation specimen and, therefore, only represent a single moment in time during a dynamic period of ontogeny for the liver.
- A single specimen cannot represent the diversity in phenotype that may result from genetic differences, sex differences, and environmental factors.
- The long period to time required to collect the data had a detrimental influence on the hepatoblast population resulting in fewer CD326^++^CD14^low^ events collected at the end of the experiment than at the beginning.

## Data Availability

The data described in this Data note can be freely and openly accessed on Flowrepository.org under “Human 21 week gestation liver phenotype (Repository ID: FR-FCM-Z58Z)” [7]. Please see Table 1 for details and links to the data, methods, and analyses.

## Abbreviations

LSECs: liver sinusoidal endothelial cells
PE: phycoerythrin
